# 2D carbon nitride as a support with single Cu, Ag, and Au atoms for carbon dioxide reduction reaction†

**DOI:** 10.1039/d3cp00392b

**Published:** 2023-02-23

**Authors:** Sergio Posada-Pérez, Anna Vidal-López, Miquel Solà, Albert Poater

**Affiliations:** a Institut de Química Computacional i Catàlisi and Departament de Química, Universitat de Girona, C/Maria Aurèlia Capmany 69 17003 Girona Catalonia Spain sergio.posada@udg.edu alber.poater@udg.edu; b Departament de Química, Universitat Autònoma de Barcelona 08193 Cerdanyola del Vallès Catalonia Spain

## Abstract

The electrochemical conversion of CO_2_ into value-added chemicals is an important approach to recycling CO_2_. In this work, we have combined the most efficient metal catalysts for this reaction, namely Cu, Ag, and Au, as single-atom particles dispersed on a two-dimensional carbon nitride support, with the aim of exploring their performance in the CO_2_ reduction reaction. Here, we report density functional theory computations showing the effect of single metal-atom particles on the support. We found that bare carbon nitride needed a high overpotential to overcome the energy barrier for the first proton–electron transfer, while the second transfer was exergonic. The deposition of single metal atoms enhances the catalytic activity of the system as the first proton–electron transfer is favored in terms of energy, although strong binding energies were found for CO adsorption on Cu and Au single atoms. Our theoretical interpretations are consistent with the experimental evidence that the competitive H_2_ generation is favored due to the strong CO binding energies. Our computational study paves the road to finding suitable metals that catalyze the first proton–electron transfer in the carbon dioxide reduction reaction and produce reaction intermediates with moderate binding energies, promoting a spillover to the carbon nitride support and thereby serving as bifunctional electrocatalysts.

## Abstract 2

Single metal atoms supported on 2D carbon nitride are promising electrocatalysts for carbon dioxide reduction reaction.

## Graphical abstract

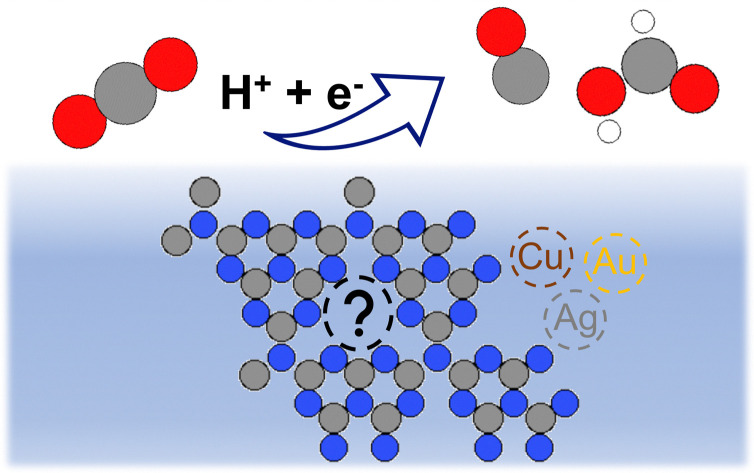

## 1. Introduction

The ever-increasing CO_2_ concentration in the atmosphere is one of the main causes of global warming.^1–3^ The Paris Agreement at COP 21 aims to limit the global peak of greenhouse gas emissions in the second half of this century. To attain this objective, CO_2_ emitted into the atmosphere must be removed. Thus, the use of technological solutions capable of storing, capturing and/or converting CO_2_ are receiving a lot of attention. CO_2_ conversion towards generating value-added compounds is becoming one of the major scientific challenges, especially in the field of heterogeneous catalysis, because a harmful byproduct like CO_2_ can be converted to an economical feedstock.^4^

The conversion of CO_2_ into valuable compounds requires a new generation of efficient and robust catalysts.^5–7^ Heterogeneous catalysis is widely used in industrial applications because of the possibility of facile separation, which reduces the operating costs, although heterogeneous catalysts often have limited selectivity. In contrast, homogeneous catalysts are very selective although they have limited industrial applications due to their cost, the use of precious metals, and the difficulty in separating and recovering the catalysts. Currently, the research community is trying to combine the properties of homogeneous and heterogeneous catalysts.^8^ From the heterogeneous catalyst perspective, research has been focused on creating smaller and dispersed catalyst particles. Single-atom catalysts (SACs), which comprise atoms of metal species dispersed on a solid support, are expected to bridge the homogeneous and heterogeneous catalyst properties.^9^ SACs can mimic a homogeneous catalyst, providing similar reactivity and product selectivity. Since the seminal work by Zhang and coworkers, who reported in 2011 that the Pt_1_/FeO_*x*_ SAC was three times more active than its nano-Pt counterpart in CO oxidation,^10^ SACs have become a new frontier in heterogeneous catalysis.

The work described herein explores the electrocatalytic CO_2_ reduction reaction (CO_2_RR) using single metal atoms (Cu, Ag, and Au) anchored in 2D graphitic carbon nitride (g-C_3_N_4_). On the one hand, we selected the group XI transition metals since they include Cu, the only transition metal capable of reducing CO_2_ to hydrocarbons and alcohols with acceptable faradaic efficiencies.^11–19^ Furthermore, the Cu–In^20,21^ and Cu–Sn^22,23^ bimetallic catalysts have been shown to selectively reduce CO_2_ to CO at low overpotentials. With respect to gold^24,25^ and silver^26^ catalysts, CO is detected as a product of the CO_2_ reduction reaction. In addition, the Cu–Ag tandem catalysts^27^ display high selectivity towards ethanol and methane production. On the other hand, g-C_3_N_4_ has emerged as a greener and low-cost material with unique attributes, such as facile synthesis, metal-free nature, earth-abundant resources, excellent thermal-physical–chemical stability, and exceptional catalytic properties.^28,29^ 2D g-C_3_N_4_ has been demonstrated to be a competitive candidate for electrocatalytic CO_2_ reduction since it can act as an active support for single metal-atom catalysts, mainly Cu, Pd, and Pt.^30–32^ Moreover, single Au atom deposition on g-C_3_N_4_ has been experimentally studied.^33^ The single metal atoms deposited on different supports are often unstable, and the isolated metal atoms tend to aggregate into nanoparticles and small clusters.^34,35^ However, g-C_3_N_4_ is an excellent substrate to stabilize metal atoms mainly due to the strong metal-nitrogen interactions. The stability and catalytic activity of the Cu/g-C_3_N_4_ system have been investigated by several authors, revealing strong copper–nitrogen interactions due to the confinement in its structural cavities.^31,36^ In these experimental studies, no copper clusters or nanoparticles were observed, suggesting the successful formation of copper single atoms. Moreover, in a very recent study, Jurado and co-workers demonstrated the stable deposition of single Rh atoms on g-C_3_N_4_ by means of experiments and theory.^37^ Dobrota *et al.* have shown the stability of single metal atoms on N_4_-graphene systems by means of density functional simulations,^38^ concluding that Cu, Ag, and Au show lower stability than other transition metals. However, the experimental studies using Cu_1_@g-C_3_N_4_ reveal its stability under electrochemical reaction conditions.^31^ Experimental studies focusing on the Cu_1_@g-C_3_N_4_ system have revealed the production of formic acid and H_2_ depending on the electrolyte solution.^31^ Furthermore, N-doped carbon nanotubes^39^ and N-doped carbon nanofibers^40^ show excellent catalytic activity toward CO_2_ reduction to produce formate (HCO_2_^−^) and CO, respectively. In addition, the anchoring of single metal atoms on familiar materials like N-doped porous carbon (M–N–C catalysts) enhances the electrocatalytic activity of these systems for CO_2_RR.^41–44^ Other supports, such as metal oxides, strongly interact with single metal atoms and small metal clusters,^45–49^ showing large catalytic activity as in the work of Zhang *et al.*,^10^ although it is important to remark that the oxygens in metal oxide materials may oxidize the single metal atoms, decreasing their catalytic activity.^50,51^

This work outlines the reaction mechanism of the two electron–proton transfers involved in CO_2_RR computed by means of periodic density functional simulations (DFT) using bare g-C_3_N_4_ and single Cu, Ag, and Au atoms deposited on a 2D carbon nitride monolayer. Our work can potentially help the design of low-cost catalysts by avoiding the use of pure transition metals (mainly Cu) and combining the excellent catalytic properties of precious metal catalysts at single metal atom sites using 2D carbon nitrides.

## 2. Computational details

The periodic spin-polarized DFT calculations were performed using the Perdew–Burke–Ernzerhof (PBE) exchange–correlation functional^52^ and by including the semi-empirical method of Grimme (D3) to describe the dispersion effects.^53^ The electronic density of the valence electrons was expanded onto a plane-wave basis set. The effects caused by the core electrons on the valence electrons were described using the projected augmented wave (PAW) method of Blöch^54^ as implemented by Kresse and Joubert.^55^ The threshold for electronic relaxation was less than 10^−5^ eV, and relaxation of the atomic positions was allowed until the forces acting on the atoms were always lower than 0.01 eV Å^−1^. The kinetic energy cut-off for the plane wave basis set was set to 520 eV, which has been proven enough to gain converged results for adsorbates in previous studies.^37^ All DFT simulations were performed using VASP code.^56^ Calculations that included the solvent were carried out without remarkable differences, as illustrated in Fig. S1 (ESI†), and thus, all the calculations in the main manuscript were performed without solvent effects. For example, the variation in HCOO* and COOH* binding energies on Cu_1_@C_3_N_4_ was lower than 0.01 eV when solvent effects were included, while that for CO* was 0.04 eV.

The (001) monolayer of g-C_3_N_4_ was considered (*Cmcm* space group) as it is the most favorable among those available on the materials project database.^57^ The monolayer contained 24C and 32N atoms. A Monkhorst–Pack grid^58^ of 5 × 5 × 1 *k*-points was used for integration in the reciprocal space of all surfaces. The binding energy of a single metal atom was calculated by following eqn (1),

1 *E*_ads_ = *E*_M@C_3_N_4__ − (*E*_C_3_N_4__ + *E*_M_)

where *E*_M@C_3_N_4__ is the DFT energy of the monolayer including the anchored single metal atom, *E*_C_3_N_4__ is the energy of the g-C_3_N_4_ monolayer, and *E*_M_ is the energy of one Cu, Ag, or Au atom in its ground state.

The two-electron reaction mechanism of CO_2_RR generally proceeds in two steps (eqn (2)–(4)), with different possible intermediates and products.2
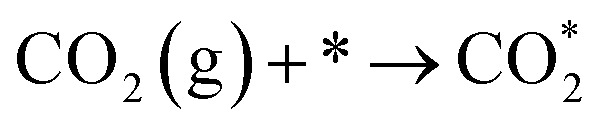
3a
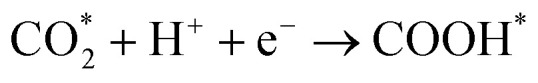
3b
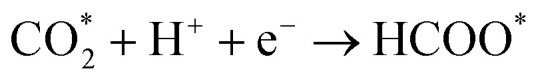

4a COOH*/HCOO* + H^+^ + e^−^ → CO* + H_2_O(g)

4b COOH*/HCOO* + H^+^ + e^−^ → HCOOH(g)

4c CO* → CO(g) + *

where * represents the g-C_3_N_4_ monolayer with and without anchored metals, and 
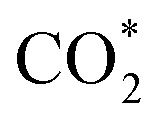
, COOH*, HCOO*, and CO* represent the adsorbed species on M@g-C_3_N_4_. According to the computational hydrogen electrode (CHE) scheme proposed by Norskov and coworkers,^59^ it is commonly assumed that the energy of H^+^ + e^−^ can be computed as half the Gibbs energy of the H_2_ molecule at 0 V *vs.* SHE. The Gibbs energy of each reaction can be obtained by computing the binding energies of the adsorbed and gas phase species. The vibrational frequencies of the adsorbed and gas phase reactants were calculated to account for zero-point energy and entropic effects. Constrained dynamics were employed while computing adsorbates and the anchored metal atom by following the same scheme used for the Rh/C_3_N_4_ system.^37^ Using this methodology, one can evaluate the Gibbs energies of the species involved in the reaction network. The computed energies can be directly related to the electrode potential, as shown in eqn (4), which demonstrates reaction 3b as an example:5
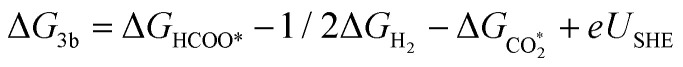
where *e* is the charge of the transferred electron, and *U* is the voltage. The maximum potential required is the electrode potential, at which all the reaction steps are energetically favored, *i.e.*, the reaction path is exergonic. Thus, the maximum potential is the Gibbs energy of the rate-limiting step. The overpotential required is the difference between the equilibrium potential of this reaction (−0.12 V) and the limiting potential. More accurate predictions of the overpotentials implied the calculation of the energy barrier of each step in the reaction mechanism along the pathway. Nevertheless, the electrochemical proton-transfer barriers have been previously shown to be very small, for instance, using Pt as the catalyst.^60,61^

## 3. Results

### 3.1 Surface corrugation and M deposition

**Fig. 1:**
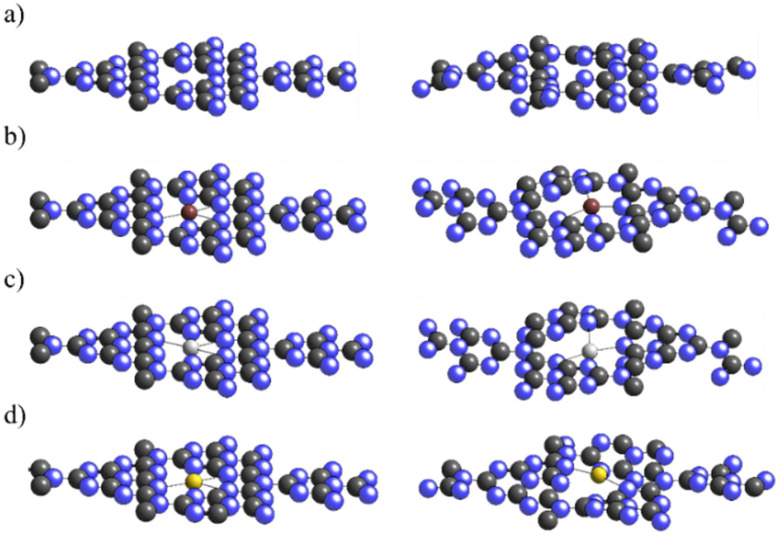
Sketches of the planar (left) and bent (right) g-C_3_N_4_ monolayers: (a) bare surface, (b) Cu_1_@g-C_3_N_4_, (c) Ag_1_@g-C_3_N_4_, and (d) Au_1_@g-C_3_N_4_.

The deposition of Cu, Ag, and Au single metal atoms was explored on the g-C_3_N_4_ (001) monolayer. The adsorption at the center site was energetically favored over adsorption at the 6-fold cavity, closing the 6-membered ring. Initially, the deposition of metal atoms did not modify monolayer planarity (see Fig. 1). Nevertheless, during the adsorption of the reaction moieties, a slight surface bending was observed due to the surface–adsorbate interaction. To further investigate the stability of this slightly bent monolayer, the structure was calculated by removing the adsorbate. From the point of view of surface energy, the corrugated surface had a monolayer stability close to 2 eV, either by clean or metal-deposited monolayer. Monolayer corrugation occurs in order to minimize the electronic repulsions experienced by the nitrogen lone pairs located in the structural holes. This implies, on the one hand, the stabilization of the Fermi level and the generation of active catalytic sites.^62^ Therefore, we considered the energy of the bent monolayer with and without the anchored single metal atoms as references. The same process was performed after including the single metal atoms, as observed in Fig. 1. The binding energies for Cu, Ag, and Au deposition were −1.17 eV, −0.84 eV, and −1.91 eV, respectively, revealing attractive and favorable interactions. With respect to stability under electrochemical conditions, the adsorption of H is considered one of the key factors. In our system, the adsorption of H was slightly repulsive for Cu and Ag (0.29 and 0.72 eV, respectively) while it was highly favored for Au (−0.92 eV); therefore, the Au sites are probably covered by H atoms under electrochemical conditions.

### 3.2 CO_2_ adsorption on bare g-C_3_N_4_ and M_1_@g-C_3_N_4_

The first step that determines the electrocatalytic reduction of CO_2_ is its adsorption on the M_1_@g-C_3_N_4_ support. As mentioned above, the adsorption process is usually omitted to compute the CO_2_RR profile using the CHE model, thus passing directly from the gas phase molecule to the COOH/HCOO adsorbed species. Nevertheless, the adsorbate–surface interactions can be large, which may hinder CO_2_ conversion. The adsorption of CO_2_ was studied on a clean monolayer (through the interaction with an N and a C atom on the g-C_3_N_4_ surface) and on top of the single metal atom. As reported in Table 1, the energy of adsorption, which takes place through the C atom of CO_2_, reached values between −0.24 and −0.28 eV. The similar binding energies point to the fact that CO_2_ maintains the gas-phase geometry after adsorption since the C=O bond length reported in Table 1 is the same, and the molecule is not bent.

**Table 1:** Binding energy (*E*_ads_), C=O distance (*d*(C–O)), and angle of CO_2_ (α(OCO)) adsorbed on the bent bare and M_1_@g-C_3_N_4_ surfaces

Surface	*E*_ads_ (eV)	*d*(C–O) (Å)	*α*(OCO) (°)
g-C_3_N_4_	−0.27	1.177/1.176	178
Cu@g-C_3_N_4_	−0.28	1.176/1.178	179
Ag@g-C_3_N_4_	−0.22	1.176/1.178	179
Au@g-C_3_N_4_	−0.24	1.175/1.178	178

### 3.3 CO_2_RR on bare g-C_3_N_4_

In this work, we have explored the formation of formic acid and carbon monoxide in a two-electron–proton transfer reaction. The first reaction step involves the formation of the carboxyl (COOH) and formate (HCOO) intermediates, which can evolve into CO and H_2_O or HCOOH after the second electron–proton transfer step. This reaction pathway competes directly with the hydrogen evolution reaction. According to the literature, the binding energy of the reaction intermediates determines the favored reaction path.^63–65^ The interaction of the gas-phase species with the support is commonly neglected when applying the CHE model. In this work, we have computed the CO_2_RR reaction mechanism with and without considering the binding energies of CO_2_, CO, and HCOOH on the M_1_@g-C_3_N_4_ surface.

**Fig. 2:**
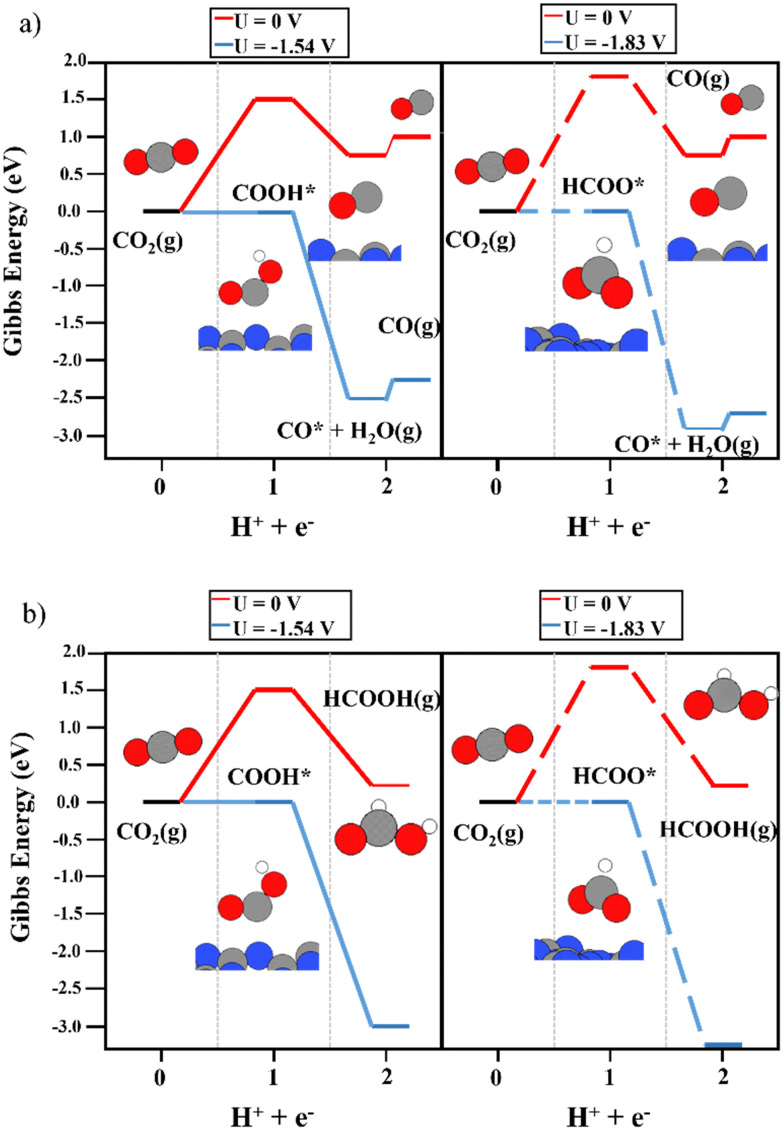
The reaction mechanism of CO_2_RR on the bare g-C_3_N_4_ monolayer at 0 V (red), and the limiting potential required to overcome the thermodynamic barriers (blue), showing (a) the formation of CO and (b) HCOOH production. The left and right panels illustrate the energy diagrams considering the formation of COOH (solid lines) and HCOO (dashed lines) as intermediates, respectively.

First of all, the electrocatalytic activity of the bare support was investigated. The conversion of CO_2_ to COOH and HCOO intermediates was not favored on the clean g-C_3_N_4_ monolayer. As shown in Fig. 2, the first electron–proton transfer required a potential higher than −1.5 V according to the CHE model, with the formation of COOH being slightly more favorable than that of HCOO. Interestingly, the second electron–proton transfer (eqn (3a) and (3b)) was exergonic. The generation of CO* + H_2_O(g) from both the COOH and HCOO intermediates (Fig. 2a) was favored by more than 0.80 eV. In addition, the desorption of CO only required 0.18 eV, showing very weak interaction with the support. Regarding HCOOH production from the COOH and HCOO moieties (Fig. 2b), the DFT simulations showed that this process was (1.65 eV) favored in terms of energy. Thus, the first electron–proton transfer is the rate-limiting step of the reaction due to the large overpotential required to generate COOH and HCOO. Fig. S2 (ESI†) shows the reaction mechanism considering the 
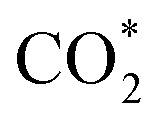
 and HCOOH* interactions with the support. The potential required to produce CO and/or HCOOH increased to compensate for the adsorption energy of 
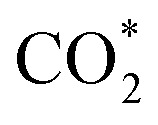
. On the other hand, to include the Gibbs energy of the formic acid molecule adsorbed on the support helped to analyze the desorption energy of the process, sorting the contribution of HCOOH generation from the HCOO* and COOH* intermediates and its desorption energy. The second electron–proton transfer implies the formation of adsorbed HCOOH*, and the process was exergonic by 1.77 eV (HCOO) and 1.35 eV (COOH). The HCOOH* desorption then only needed 0.19 eV. Despite these excellent and promising results for the second proton–electron transfer reaction, including very low desorption energies of both products, the first proton–electron transfer step hinders the catalytic activity of the bare g-C_3_N_4_ monolayer.

### 3.4 CO_2_RR on M_1_@g-C_3_N_4_

**Fig. 3:**
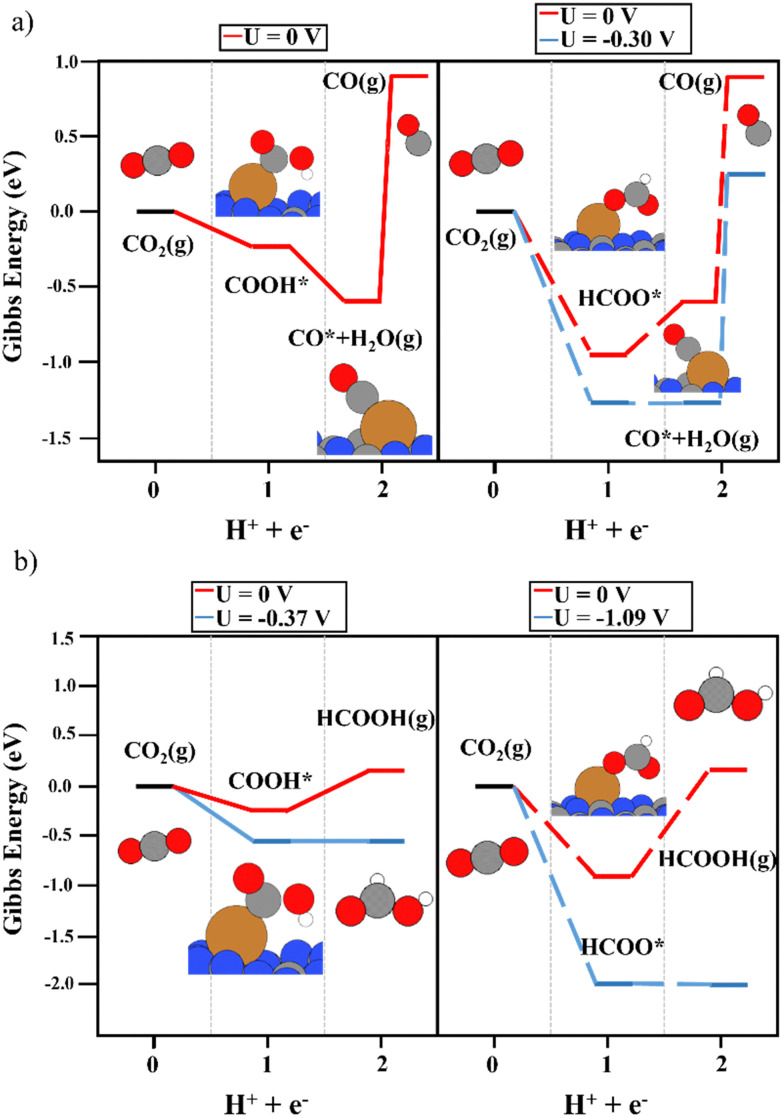
The reaction mechanism of CO_2_RR on the Cu_1_@g-C_3_N_4_ monolayer at 0 V (red) and the limiting potential required to overcome the thermodynamic barriers (blue), showing (a) the formation of CO and (b) HCOOH production. The left and right panels illustrate the energy diagrams considering COOH (solid lines) and HCOO (dashed lines) as intermediates, respectively.

**Fig. 4:**
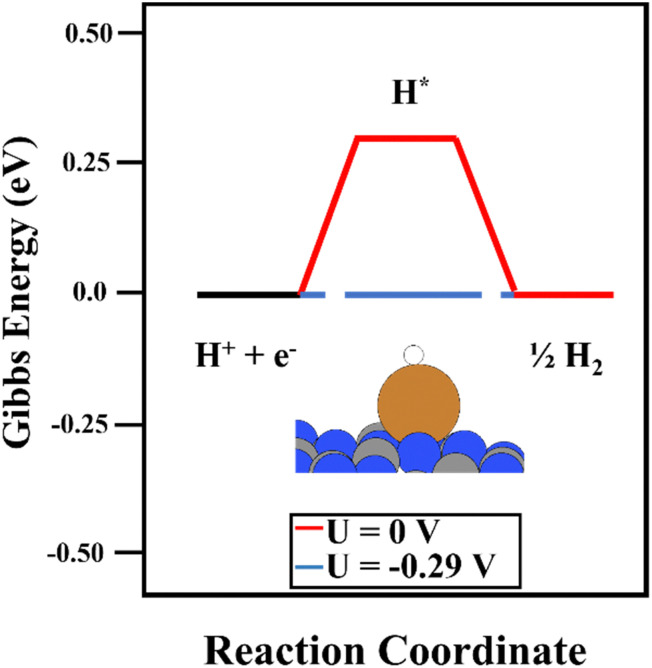
The reaction mechanism of HER on the Cu_1_@g-C_3_N_4_ monolayer at 0 V (red) and the overpotential required to overcome the thermodynamic barriers (blue).

The deposition of single metal atoms drastically modifies the electrocatalytic activity of the system. As for the Cu atom, the conversion of CO_2_ to both COOH and HCOO intermediates was energetically favored (see Fig. 3), in contrast to the results obtained on the bare support. The binding energy of HCOO (Fig. 3a and b, right panels) was larger than that of COOH (Fig. 3a and b, left panels). This is not unexpected since HCOO is typically observed in CO_2_ hydrogenation using Cu-based catalysts.^50,66–68^ This strong interaction required a limiting potential of −0.30 V to undergo the second electron–proton transfer process to produce CO, whereas, the moderate interaction of the carboxyl moiety with the Cu atom favored the second reaction step without an overpotential. Thus, an overpotential of 0.42 was required. The difference in the binding energies of both reaction intermediates can be attributed to the adsorption mode. As depicted in Fig. 3, HCOO is adsorbed in a bidentate mode, with both O atoms in contact with the Cu single atom, while in the case of COOH, the intermediate is adsorbed *via* the C atom. This leads to the large binding energy of HCOO. Including the binding energy of CO_2_ with the Cu atom in the CHE model (see Fig. S3, ESI†), a limiting potential of 0.1 V is required. However, one important shortcoming was observed. The binding energy of CO on a Cu single atom was strong (1.54 eV), making the desorption process the rate-limiting step of the reaction. Thus, to release CO from the electrocatalytic active site, more electron–proton transfer steps are required to promote CO conversion. It is important to mention that metals that bind CO strongly produce few CO_2_RR products, and consequently, the competing water reduction reaction can produce H_2_ as the main product.^69^Fig. 3b depicts the reaction mechanism of HCOOH production, where the COOH intermediate favors its formation compared with HCOO. A limiting potential of −0.37 V is predicted to make the second electron–proton step thermodynamically feasible since the first protonation is energetically favored. As illustrated in Fig. S3 (ESI†), the COOH* + H^+^ + e^−^ → HCOOH* reaction step is isoenergetic, *i.e.* the production of formic acid adsorbed on top of a Cu single atom does not require an overpotential. The potential required to generate HCOOH (g) (0.37 eV) is linked with HCOOH desorption. Again, the strong binding energy of the HCOO moiety in a bidentate adsorption mode on top of the Cu atom makes the formation of HCOOH difficult since the threshold of the second electron–proton transfer step is 1.09 eV higher in energy. In contrast to the observations using the COOH intermediate, the HCOO* + H^+^ + e^−^ → HCOOH* step was not isoenergetic but endergonic by 0.73 eV (Fig. S3, ESI†). In summary, our simulations show that Cu_1_@g-C_3_N_4_ catalyzes the first proton–electron transfer, favoring the formation of COOH and HCOO, with the latter showing the largest binding energy with the Cu atom. This fact implies a large overpotential for the second proton–electron transfer step. Nevertheless, the production of CO and HCOOH from the COOH intermediate is favored. Furthermore, the binding energy of CO hinders its desorption and can enhance the formation of C2 compounds after more proton–electron transfer steps or promote the competitive H_2_ generation (HER) reaction. To further explore the HER vs CO_2_RR competition, the energy profile of HER was obtained, as illustrated in Fig. 4. One can observe that an overpotential of 0.29 V is required to produce H_2_. Indeed, CO production is energetically favored, although the strong Cu–CO interaction prevents its desorption. In contrast, the H_2_ molecule binds weakly to Cu, at only 0.42 eV, and the overpotential required is slightly lower than that needed for the formation of HCOOH. Our DFT results agree with experimental studies, wherein only H_2_ and HCOOH were found as products.^31^ On the one hand, the large binding energy of CO with the Cu single atom prevents its production, favoring the hydrogen evolution reaction. On the other hand, our calculations predict the facile formation of formic acid by means of COOH due to its moderate binding energy with Cu and the energetically favored proton–electron transfer step to generate HCOOH.

**Fig. 5:**
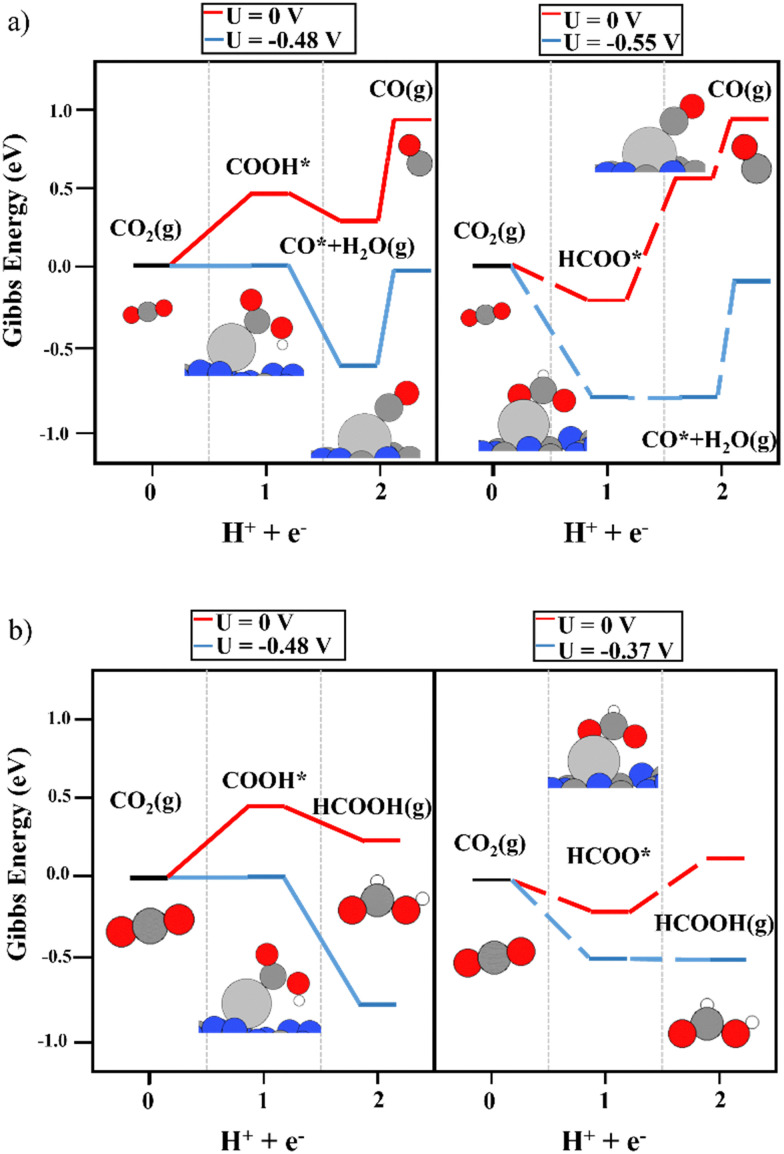
The reaction mechanism of CO_2_RR on the Ag_1_@g-C_3_N_4_ monolayer at 0 V (red) and the limiting potential required to overcome the thermodynamic barriers (blue), showing (a) the formation of CO and (b) HCOOH production. The left and right panels illustrate the energy diagrams considering COOH (solid lines) and HCOO (dashed lines) as intermediates, respectively.

The use of Ag as a single-atom catalyst presented different activity than that of Cu. Regarding the first proton–electron transfer, the formation of HCOO was energetically favored, as reported for the Cu single atom, whereas, an overpotential was required to generate COOH (see Fig. 5a). Therefore, the discussion is focused on the second proton-transfer step starting from the HCOO intermediate (the right panels in Fig. 5a and b). The production of CO from HCOO was endergonic, and consequently, a limiting potential of −0.55 V was needed to overcome the thermodynamic barrier. Although the desorption energy of CO was around 1 eV lower than the energy predicted using a Cu single atom, it was still energetically demanding (0.56 eV). The production of formic acid, as reported in Fig. 5b, was the most favored reaction pathway since it required only a potential of −0.37 V, with the second proton–electron transfer step being the most energy-demanding. Regarding the possible competition of HER, in this case, the *H*_ads_ on top of Ag was unstable at 0.72 eV, which is a higher overpotential than that required for CO_2_RR. To conclude, the Ag_1_@g-C_3_N_4_ system promotes the production of HCOOH, with a slightly lower overpotential than that of the formation of CO. In addition, when the adsorption energy of HCOOH was included in the CHE model, it could be observed that the HCOO* + H^+^ + e^−^ → HCOOH* reaction required only −0.17 V, whereas the desorption energy of adsorbed HCOOH was 0.19 eV, which is lower than the desorption energy of the CO molecule on top of the Ag single atom (see Fig. S4, ESI†).

**Fig. 6:**
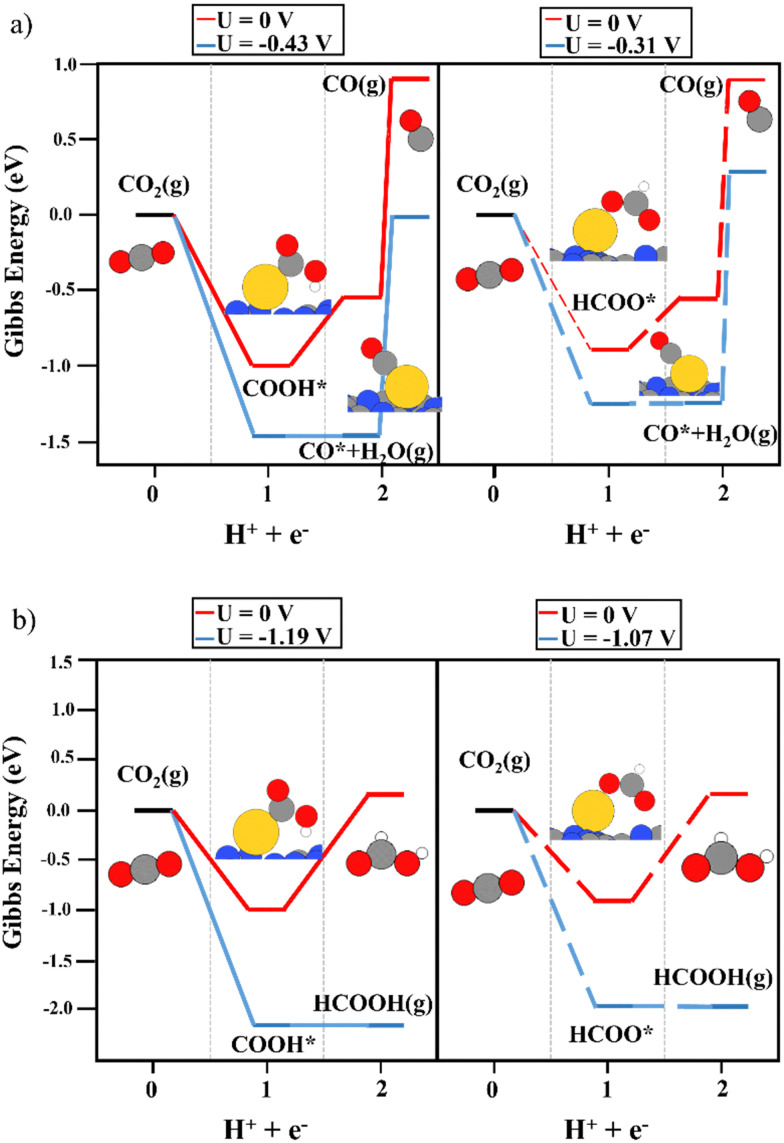
The reaction mechanism of CO_2_RR on the Au_1_@g-C_3_N_4_ monolayer at 0 V (red) and the limiting potential required to overcome the thermodynamic barriers (blue), showing (a) the formation of CO and (b) HCOOH production. The left and right panels illustrate the energy diagrams considering COOH (solid lines) and HCOO (dashed lines) as intermediates, respectively.

Prior to analyzing the results of Au_1_@g-C_3_N_4_, the large binding energy of the H atoms on top of the Au atom must be evaluated. It may imply that the catalytic active site is covered by H atoms at operational conditions, which can poison the single Au atom site. However, this does not mean that Au favors the HER since the binding energy is too large to favor H_2_ production. Focusing on the results of CO_2_RR (see Fig. 6), the first proton–electron transfer was exergonic, with COOH (−1.00 eV) being a slightly more favored intermediate than HCOO (−0.88 eV), which is in contrast to Cu and Ag. Though the exergonicity of this step was also observed over the Cu_1_@g-C_3_N_4_ catalyst, in the Cu system, the formation of HCOO is clearly favored. In the Au_1_@g-C_3_N_4_ catalyst, the interaction of both intermediates with the anchored Au atom was strong. For the second proton–electron transfer, a lower overpotential was required to produce CO (Fig. 6a) rather than HCOOH (Fig. 6b). Nevertheless, as observed for Cu_1_@g-C_3_N_4_, the desorption of CO was not feasible due to the strong interaction with the Au atom (1.50 eV). According to experiments using a Cu single atom on C_3_N_4_, this strong binding energy favors H_2_ generation.^31^ Furthermore, our simulations rule out the *a priori* production of HCOOH due to this large overpotential. On the other hand, we computed the reaction mechanism considering HCOOH adsorption in the CHE model. As illustrated in Fig. S5 (ESI†), a large overpotential was required to perform the second electron–proton transfer (COOH*/HCOO* + H^+^ + e^−^ → HCOOH*). In the case of the Cu_1_@g-C_3_N_4_ system, this process was isoenergetic, and the main shortcoming was the HCOOH desorption energy. However, when using Au instead of Cu, this process is not feasible. The adsorption of HCOOH* is less favored on top of Au.

In summary, Cu and Au single-metal atoms anchored on g-C_3_N_4_ catalyze the first electron–proton transfer to generate COOH and HCOO as intermediates without an overpotential, while Ag only favors the formation of HCOO. The main drawback of these systems is the strong binding energy of the metal with the HCOO intermediate and CO, which implies the need for large overpotentials, and the strong Au–H interaction. For HCOOH production from HCOO and COOH intermediates, Ag_1_@g-C_3_N_4_ and Cu_1_@g-C_3_N_4_ require only a limiting potential of −0.37 V. With respect to CO generation, the Cu_1_@g-C_3_N_4_ system does not require an overpotential although the strong interaction of CO with Cu (also observed with Au) hinders its production. Finally, we have evaluated the role of the 2D carbon nitride on the CO_2_RR. Large overpotentials are required to overcome the thermodynamic barrier of the first electron–proton transfer, whereas it is significant that the second transfer is exergonic. This opens the possibility for M_1_@g-C_3_N_4_ to function as bifunctional catalysts, where the single metal atoms are responsible for the first electron–proton transfer to generate the carboxyl and formate intermediates, and after the HCOO and COOH spillover to the g-C_3_N_4_ monolayer, HCOOH and CO production becomes feasible. The strong interaction of CO with single metal atoms probably will hinder its diffusion to the surface, promoting the competitive reaction, namely H_2_ generation.

## 4. Conclusions

The computational hydrogen electrode model has been used to explore the suitability of Cu, Ag, and Au single atoms supported on 2D carbon nitride as electrocatalysts for CO_2_RR. Our DFT simulations reveal that bare g-C_3_N_4_ is a good electrocatalyst for the second electron–proton transfer to generate CO and HCOOH, although according to the calculations, the initial conversion of CO_2_ to COOH and/or HCOO moieties requires a very large overpotential.

The deposition of single metal atoms changes the rate-limiting step of the reaction. The Cu, Ag, and Au single atoms carry out the first proton–electron transfer without an overpotential (except for Ag to generate the COOH intermediate). Nevertheless, the drawbacks of these systems are linked to the strong interaction of HCOO and CO with the single-metal atoms. The Cu_1_@g-C_3_N_4_ system can produce CO without an overpotential and HCOOH with a low limiting potential of −0.37 V. However, HCOO formation is preferred due to its strong interaction with Cu, which hinders further HCOO conversion towards CO and/or HCOOH. The carboxyl intermediate shows a moderate binding energy and helps the production of CO, although a desorption energy higher than 1.50 eV is predicted, which can make its production difficult. Moreover, the overpotential required for HER is slightly lower, and as confirmed by experiments, can promote the HER instead of CO_2_RR. The Ag_1_@g-C_3_N_4_ system promotes the formation of the HCOO intermediate. The Au_1_@g-C_3_N_4_ system exhibits the same drawback as Cu_1_@g-C_3_N_4_, with a similar desorption energy for CO, and slightly large overpotentials with respect to the Cu and Ag systems to produce HCOOH. Furthermore, Au shows large binding energy toward H atoms, which can block the active surface sites. Taken together, our work suggests that these systems may work as bifunctional catalysts, with the metal atom being responsible for the first electron–proton transfer and the support facilitating the second transfer and product desorption.

## Conflicts of interest

There are no conflicts to declare.

## Supplementary Material

CP-025-D3CP00392B-s001
