# Supplementary material for: 2D carbon nitride as a support with single Cu, Ag, and Au atoms for carbon dioxide reduction reaction
Source: Phys Chem Chem Phys. 2023 Feb 23;25(12):8574–82. doi: 10.1039/d3cp00392b (PMC10277901; doi:10.1039/d3cp00392b)
Supplement: CP-025-D3CP00392B-s001 [file CP-025-D3CP00392B-s001.pdf]

Electronic Supplementary Material (ESI) for Physical Chemistry Chemical Physics.
This journal is © the Owner Societies 2023

Supporting Information

2D Carbon Nitride as Support of Single Cu, Ag, and Au Atoms for Carbon Dioxide Reduction Reaction

Sergio Posada-Pérez,[a*] Anna Vidal-López,[a,b] Miquel Solà,[a] and Albert Poater[a*]

[a]Institut de Química Computacional i Catàlisi and Departament de Química, Universitat de Girona, c/ Maria Aurèlia Capmany 69, 17003 Girona, Catalonia, Spain.
[b] Departament de Química, Universitat Autònoma de Barcelona, 08193 Cerdanyola del Vallès, Catalonia, Spain.
Corresponding author: Sergio Posada-Pérez (sergio.posada@udg.edu), Albert Poater (alber.poater@udg.edu)

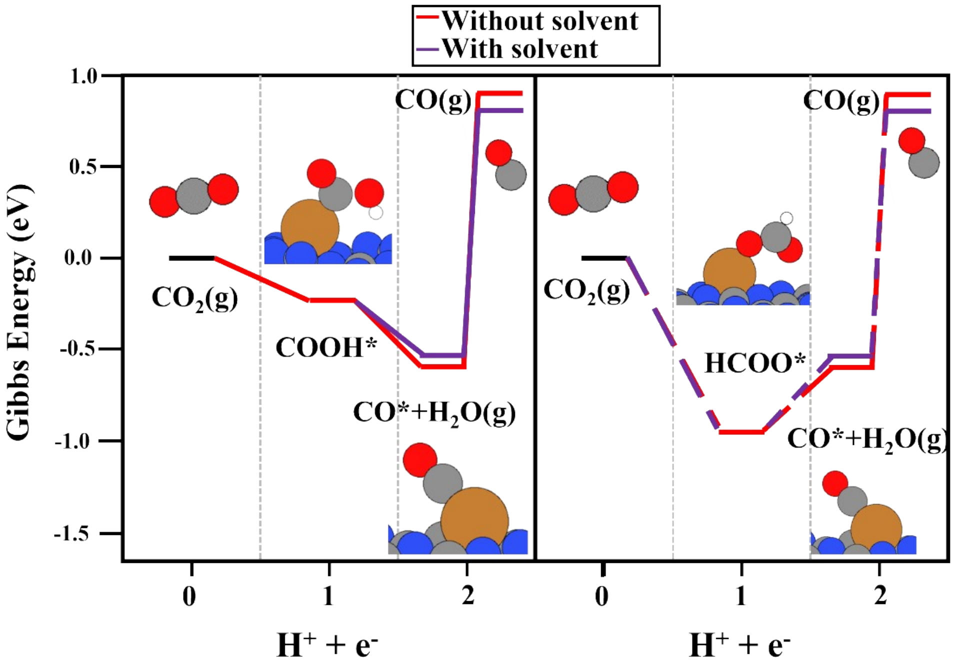

Figure S1: Reaction mechanism of CO_2RR on bare $Cu_1@g\text{-}C_3N_4$ considering VASP calculations with and without solvent effects.

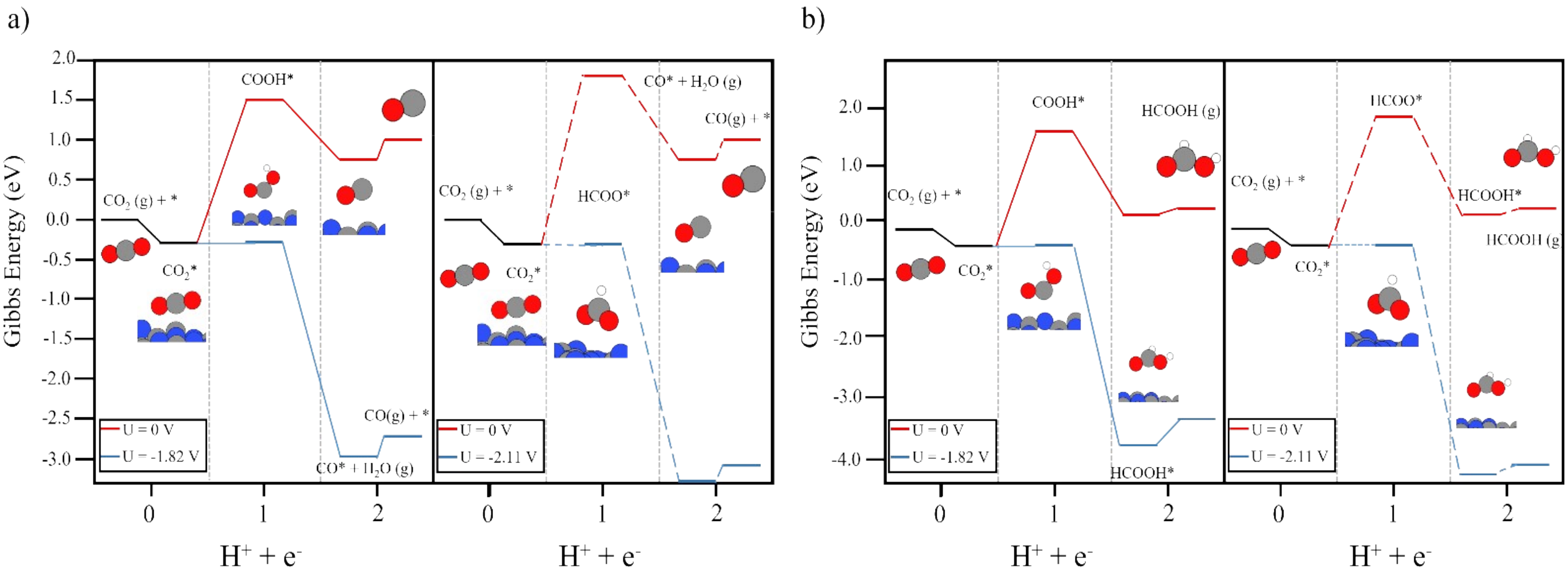

Figure S2: Reaction mechanism of CO_2RR on bare g-C_3N_4 monolayer at 0V (red) and the limiting potential required to overcome the thermodynamic barriers (blue). These plots include the effect of CO_2 and HCOOH adsorption energy, showing (a) the formation of CO and (b) the HCOOH production. Left and right panels illustrate the energy diagram considering the COOH (solid lines) and HCOO (dashed lines) intermediates, respectively.

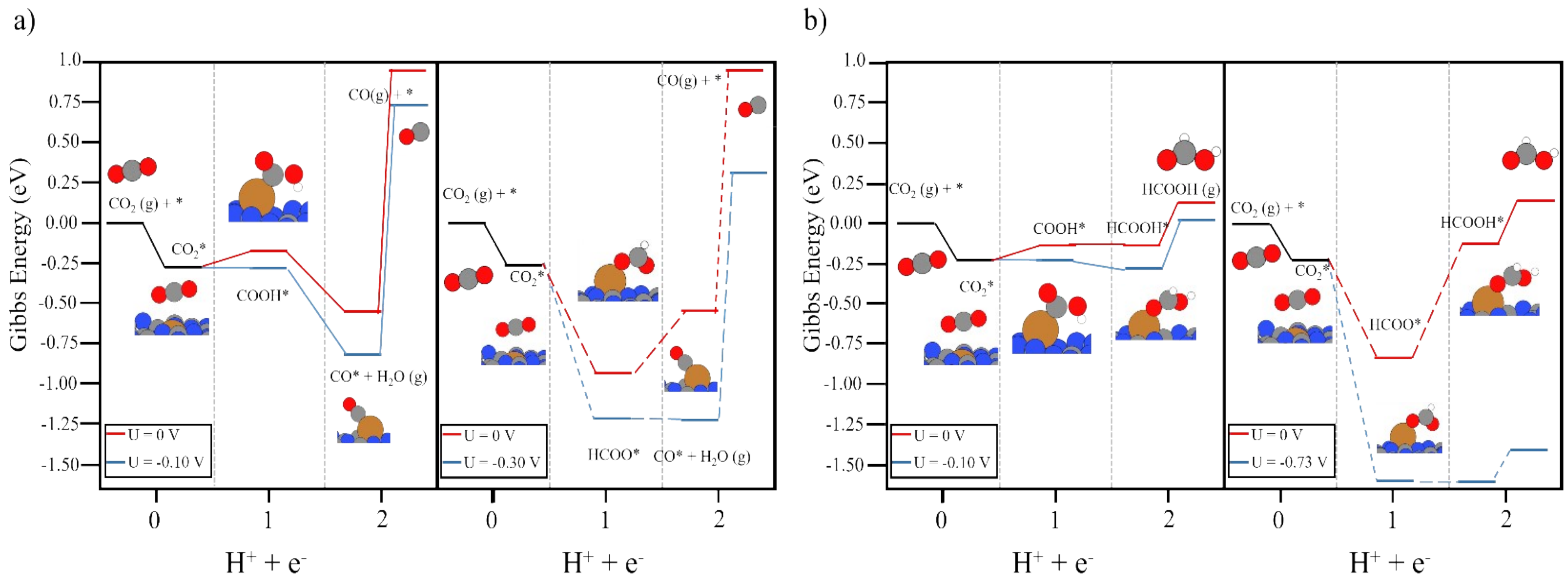

Figure S3: Reaction mechanism of CO_2RR on $Cu_1@g\text{-}C_3N_4$ monolayer at 0V (red) and the limiting potential required to overcome the thermodynamic barriers (blue). These plots include the effect of CO_2 and HCOOH adsorption energy showing (a) the formation of CO and (b) the HCOOH production. Left and right panels illustrate the energy diagram considering the COOH (solid lines) and HCOO (dashed lines) intermediates, respectively.

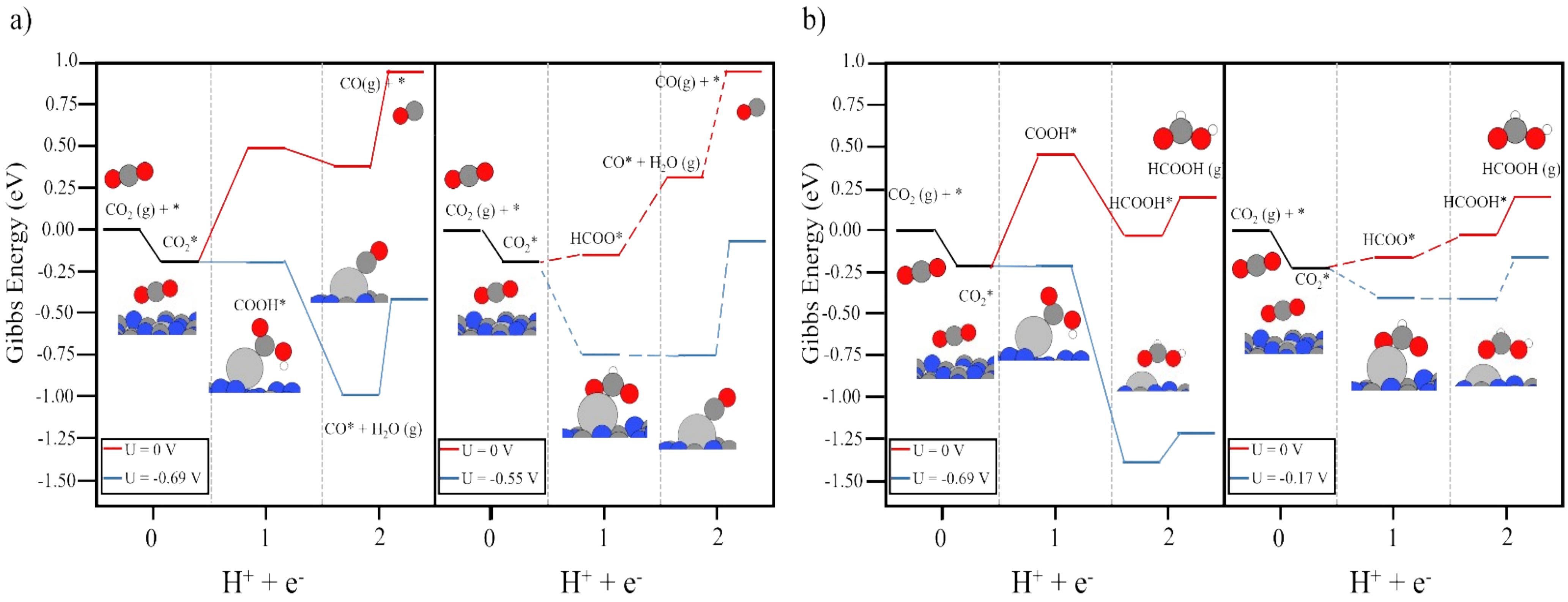

Figure S4: Reaction mechanism of CO_2RR on Ag_1@g-C_3N_4 monolayer at 0V (red) and the limiting potential required to overcome the thermodynamic barriers (blue). These plots include the effect of CO_2 and HCOOH adsorption energy showing (a) the formation of CO and (b) the HCOOH production. Left and right panels illustrate the energy diagram considering the COOH (solid lines) and HCOO (dashed lunes) intermediates, respectively.

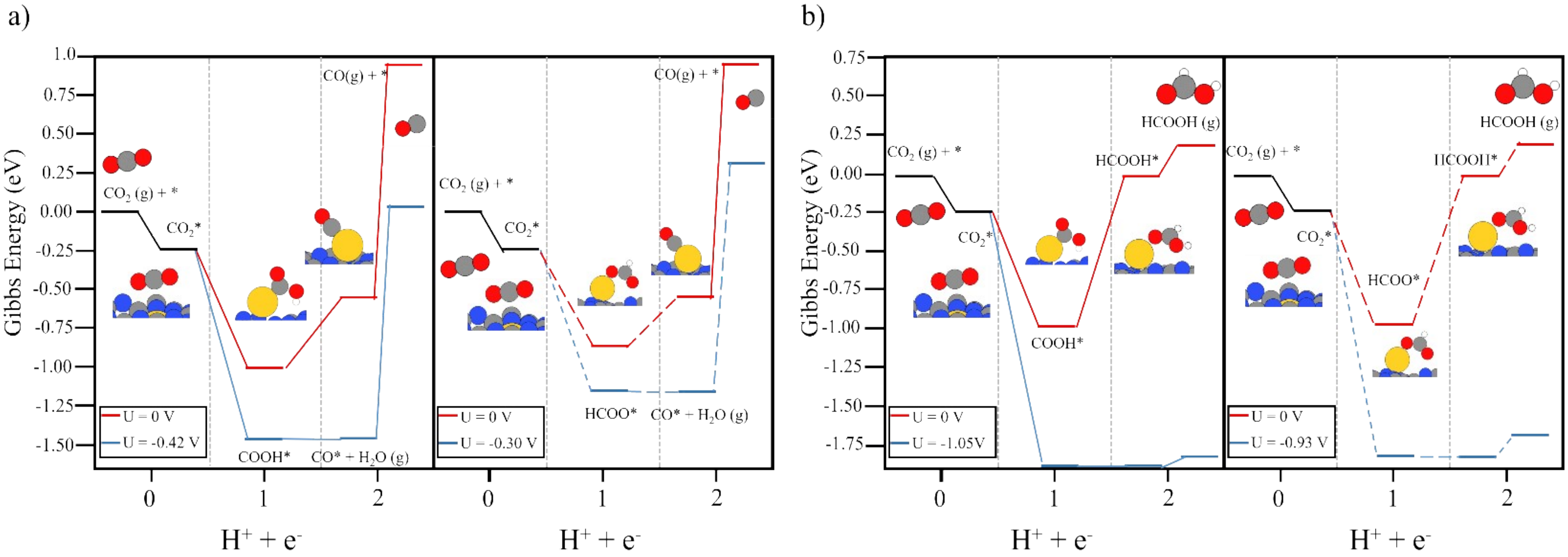

Figure S5: Reaction mechanism of CO_2RR on Au_1@g-C_3N_4 monolayer at 0V (red) and the limiting potential required to overcome the thermodynamic barriers (blue). These plots include the effect of CO_2 and HCOOH adsorption energy showing (a) the formation of CO and (b) the HCOOH production. Left and right panels illustrate the energy diagram considering the COOH (solid lines) and HCOO (dashed lines) intermediates, respectively.
